# Blood Donation Practices and Awareness of Blood Types Among Adults in the United Arab Emirates: A Cross-Sectional Community-Based Study

**DOI:** 10.7759/cureus.52044

**Published:** 2024-01-10

**Authors:** Dima Saleh, Ghaith AlWawi, Rand Tayyem, Alaa Al Hajji, Reem Alketbi, Majd Albeetar

**Affiliations:** 1 Department of Medicine, Dubai Academic Health Corporation, Dubai, ARE; 2 Department of Medicine, Emirates Health Services, Sharjah, ARE; 3 Department of Medicine, Burjeel Medical City, Abu Dhabi, ARE; 4 Department of Medicine, University of Sharjah, Sharjah, ARE; 5 Department of Medicine, Saudi German Hospital, Dubai, ARE

**Keywords:** united arab emirates, blood donation, public health, blood transfusion, blood types

## Abstract

Background

Blood donation serves as a crucial component of healthcare systems worldwide, ensuring the availability of blood for life-saving therapies and medical procedures. To date, no studies have assessed the knowledge of blood donors regarding their own blood type, overall awareness of blood types, and blood donation practices within the United Arab Emirates (UAE) population.

Aim

The present study's primary objective is to assess knowledge regarding blood types and blood donation practices among adult blood donors in the UAE. The aim is to evaluate the effectiveness of existing initiatives and propose targeted strategies to recruit new donors and retain existing ones, with a specific emphasis on less common or critical blood types like O-negative.

Methods

This cross-sectional community-based study involved 259 participants selected through convenience sampling. Interviews were conducted at blood donation sites, and blood type data for each participant was collected from the blood bank following their donation. Statistical analysis was conducted using SPSS Statistics version 25 (IBM Corp. Released 2017. IBM SPSS Statistics for Windows, Version 25.0. Armonk, NY: IBM Corp.), and p-values of <0.05 were considered and reported as statistically significant.

Results

Blood type distribution showed a prevalence of O+ (41.7%), A+ (22.4%), and B+ (19.7%). Notably, the rarest blood types observed were O- (2.3%) and AB- (0.4%). First-time blood donors comprised 29.7%, while the rest had previously donated blood at least once. About 72.2% of participants identified their blood type correctly, but knowledge regarding the concept of blood compatibility was limited. Emirati nationality, higher education level, and a history of previous blood donations were significantly associated with blood type awareness. Perceived accessibility to blood donation was generally high. The most reported motivational drives for blood donation included awareness of blood donation significance, a sense of ethical duty, and religious beliefs.

Conclusion

Most participants correctly identified their blood type; awareness was associated with Emirati nationality, higher educational achievement, and a history of previous blood donation. The high perceived accessibility of blood donation, coupled with the observed prevalence of previous blood donations among participants, offers valuable feedback on the current initiatives in the UAE for blood donation. Our study identifies target demographics and motivational triggers, offering valuable insights for optimizing blood donation initiatives in the UAE. These findings provide a strategic foundation for enhancing awareness and participation in this vital healthcare domain.

## Introduction

Background information

Blood transfusion is a cornerstone in modern medical practice, and the compatibility of blood types is paramount to its success [1,2]. In the realm of transfusion medicine, the O-negative blood type holds a unique position due to its universal donor status. In emergent situations where time is of the essence, O-negative blood can be transfused to any patient, regardless of their blood type, rendering it invaluable in resuscitative efforts [1,3]. Consequently, the demand for O-negative blood continually surpasses its supply, often resulting in critical shortages that can jeopardize patient care [4,5].

Globally, the importance of O-negative blood is acknowledged; however, its significance becomes even more pronounced in countries with heightened emergency medical scenarios [3]. For instance, in the United Arab Emirates (UAE), trauma is the second leading cause of death, leading to an amplified demand for emergency medical services and, by extension, O-negative blood [6,7].

While multiple studies have assessed the awareness and knowledge of blood types and blood donation in the Arab Gulf region, most targeted specialized groups such as university or healthcare staff and students, compromising the generalizability of the results to the general public. There are limited studies that assess awareness in the general population [8,9]. To our knowledge, no studies have been conducted in the UAE assessing the general public's knowledge and awareness of blood types and blood donation practices. This study seeks to address this gap by evaluating knowledge and awareness of blood types and blood donation practices among adult blood donors in the UAE.

Significance of the study

Awareness of one's blood type could potentially serve as a catalyst to boost blood donation drives [10,11]. Individuals knowledgeable of their blood type might feel more inclined to donate, thereby contributing to blood bank reserves [12]. In the UAE, where cultural and societal norms may influence blood donation tendencies, emphasizing the importance of blood type knowledge could be transformative [13].

Moreover, this study holds particular relevance to the UAE due to its distinctive demographic mosaic, accommodating both locals and non-locals [14]. This diverse blend may lead to varying perceptions and awareness levels about blood types, donation habits, and overall health behaviors. By exploring awareness levels in this setting, the findings could be pivotal in strategizing effective blood donation campaigns tailored for the UAE population.

Research objective

Understanding awareness levels about blood types is crucial, given their potential influence on blood donation tendencies [15]. In many societies, heightened personal health literacy, including knowledge of one's blood type, serves as a precursor to active participation in healthcare activities, such as blood donation [16]. The primary objective of this study is to assess the knowledge of the UAE’s adult population of blood donors about their own blood types and evaluate their awareness of the concept of blood compatibility. It also provides valuable insights into the prevalence of each blood type in the studied sample, investigates sources of information used by participants, and explores potential associations between knowledge and demographic factors. The findings could establish a foundational knowledge base for healthcare policymakers and campaign planners, aiding efforts to enhance blood donation rates and overall healthcare outcomes in the region.

## Materials and methods

Study design

This study utilized a cross-sectional community-based design, emphasizing accessibility through a convenience sampling method. The initial target sample size, calculated using Yamane's formula, was approximately 385 with a 5% margin of error. However, logistical constraints resulted in a final sample size of 259 participants.

A comprehensive literature review was conducted to explore studies of a similar nature in the UAE and neighboring countries. Identifying a literature gap concerning knowledge and awareness of blood types, donation, and transfusion within the UAE informed the study's conceptualization. To ensure accurate data representation of our population, personal interviews conducted by the authors were employed. For assessing participants' awareness of their blood type, the blood bank's data on each individual participant's blood type was utilized, ensuring accuracy and validity in our study.

Ethical proposal and approval

Adhering to ethical standards, an official proposal was submitted to the Research Ethics Committee of Medical Colleges, University of Sharjah, and approval was obtained with the reference number REC-20-02-03-03-S. During the data collection process, all participants received a detailed information sheet clarifying the study's nature. Written consent was obtained before the interview, ensuring response confidentiality and granting permission to access information about the participant's blood type from the bank's database.

Tool development and validation

A comprehensive questionnaire was developed as the primary data collection tool for this study. Given the unique focus of our investigation, no existing tools were suitable, prompting the authors to construct a customized questionnaire. The questionnaire consisted of 21 items organized into five sections, covering demographics, knowledge and awareness, practices and views, sources of information, and the participant's blood type as reported by the blood bank.

The demographic section covered age, sex, nationality, social status, educational level, and career field. In the knowledge and awareness section, participants were queried about their awareness of their own and their partner's blood type, as well as their understanding of concepts like blood compatibility, universal donor, and universal recipient. The practices and views section explored the frequency and motives behind blood donation, participants' views on blood donation accessibility in the UAE, and opinions on documenting blood types on official documents like the Emirates ID (see Appendices section).

Given the questionnaire's broad focus, encompassing awareness, blood donation practices, motivations, perspectives, and information sources, and with only a subset of items specifically addressing participants' factual knowledge (awareness of their blood type and knowledge of universal blood donors and recipients), assigning scores categorizing participants as knowledgeable or not on these matters was deemed inappropriate. This study, therefore, serves as an examination of the attitudes of adult blood donors in the UAE toward the topic.

Following tool construction, a pilot study involving 18 participants was conducted to identify and rectify potential errors. Feedback from the pilot study informed modifications for coherence and relevance. To ensure survey comprehension and minimize errors, interviewers adhered to standardized responses, addressing potential clarification requests during the interview process. These efforts aimed to avoid providing leading responses while accurately capturing the participant's authentic perspectives.

Data collection process

Our study focused on blood donors aged 18 years or older. To evaluate eligibility for donation, all donors underwent examination by the on-site medical assessment and screening team, adhering to the World Health Organization's (WHO) international guidelines for blood donation fitness [17]. Participants who either declined to consent to the interview or were deemed medically unfit to donate blood were excluded.

Interviews took place at various blood donation sites, where each participant was assigned an identification number, formed by combining the last three digits of their phone number with their initials. Blood type results, obtained through typing and crossmatching in the blood bank labs, were matched with participant data from the interview using these identification numbers, ensuring data accuracy and traceability.

Data analysis and interpretation

Data collected was extracted and analyzed using SPSS Statistics version 25 (IBM Corp. Released 2017. IBM SPSS Statistics for Windows, Version 25.0. Armonk, NY: IBM Corp.). Descriptive statistics, means, and proportions were calculated. The significance of the results was assessed using Pearson's chi-square test, with p-values below 0.05 considered statistically significant. Descriptive statistics were used to summarize the data. The chi-square test was employed to analyze the association between blood donors' demographic information and their knowledge of blood types.

## Results

Demographics and general information

A total of 259 participants were interviewed. The average age was 36.5 years (SD=8.175), with participants ranging between 20 and 64 years. The majority of the participants were males, accounting for 82.31% (n=214), while females constituted 17.69% (n=45). The marital status distribution revealed that 79.62% (n=206) were in a relationship, while 20.38% (n=53) were single. Locals comprised a minor segment at 5.4% (n=14), with nonlocals representing 94.6% (n=245). Among the nonlocals, 41.6% (n=102) were Indian, followed by 18.4% (n=45) Bangladeshi, and 11.0% (n=27) Filipino. In terms of education, 33.2% (n=86) had qualifications below a bachelor's degree, whereas 66.8% (n=173) had a bachelor's degree or higher.

Blood type distribution

The distribution of blood types, as revealed by the blood bank's lab analysis, is presented in Table 1. Among the participants, the most prevalent blood type was O+ at 41.7% (n=108), followed by A+ at 22.4% (n=58). Other common blood types included B+ at 19.7% (n=51) and AB+ at 6.2% (n=16). A- and B-blood types were reported at 3.9% (n=10) and 3.5% (n=9), respectively. The rarest blood types among the surveyed participants were AB- at 0.4% (n=1) and O- at 2.3% (n=6).

**Table 1:** Prevalence of blood types following blood typing

Blood type	Frequency	Percentage
A+	58	22.4%
A-	10	3.9%
B+	51	19.7%
B-	9	3.5%
AB+	16	6.2%
AB-	1	0.4%
O+	108	41.7%
O-	6	2.3%

Knowledge of blood types

Of the respondents, 72.2% (n=187) were aware of their blood type and reported it correctly. In contrast, 27.8% (n=72) lacked this knowledge or reported it incorrectly. Analysis revealed no significant associations between age, sex, or occupation and blood type awareness. However, locals exhibited higher knowledge than nonlocals [X²(1,258)=5.730, p=0.013]. A direct correlation emerged between educational attainment and blood type awareness [X²(1,259)=8.835, p=0.003].

Participants with at least one previous blood donation in the past demonstrated superior knowledge compared to first-time donors [X²(1,259)=51.261, p<0.0005]. A small proportion of previous donors (5.8%, n=15) inaccurately reported their blood type, as revealed by a comparison with the blood bank's blood typing data (Table 2).

**Table 2:** Knowledge of blood types in relation to participants' demographics Statistical significance is defined by p<0.05

Variables		Frequency	Correctly reported their blood type	Unaware of their blood type	Pearson's chi-square	p-value
Age group (years)	<31	50 (19.3%)	32 (64.0%)	18 (36.0%)	X^2^(3.259) = 3.092, Df = 3	p-value = 0.378
	31-34	66 (25.4%)	46 (69.7%)	20 (30.3%)		
	35-40	73 (28.1%)	55 (75.3%)	18 (24.7%)		
	>40	70 (27%)	54 (77.1%)	16 (22.9%)		
Sex	Males	214 (82.3%)	152 (71%)	62 (29%)	X^2^(1.259) = 0.844, Df = 1	p-value = 0.358
	Females	45 (17.4%)	35 (77.8%)	10 (22.2%)		
Nationality	Locals	14 (5.4%)	14 (100.0%)	0 (0.0%)	X^2^(1.258) = 5.730, Df = 1	p-value = 0.013
	Non-locals	245 (94.2%)	172 (70.5%)	72 (29.5%)		
Marital status	Married	206 (79.2%)	152 (73.8%)	54 (26.2%)	X^2^(1.260) = 1.083, Df = 1	p-value = 0.298
	Single	53 (20.8%)	36 (66.7%)	18 (33.3%)		
Educational level	Elementary school	1 (0.4%)	1 (100.0%)	0 (0.0%)	X^2^(1.259) = 8.835, Df = 1	p-value = 0.003
	Middle school	6 (2.3%)	3 (50.0%)	3 (50.0%)		
	High school	79 (30.8%)	48 (60.8%)	31 (39.2%)		
	Technical school	6 (2.3%)	4 (66.7%)	2 (33.3%)		
	Diploma degree	26 (10%)	16 (61.5%)	10 (38.5%)		
	Bachelor's degree	107 (41.2%)	86 (80.4%)	21 (19.6%)		
	Postgraduate degree	34 (13.1%)	29 (85.3%)	5 (14.7%)		
Occupation	Medical field	14 (5.4%)	11 (78.6%)	3 (21.4%)	X2(1.254) = 0.216, Df = 1	p-value = 0.765
	Other	245 (92.7%)	175 (73.0%)	70 (27.0%)		
History of previous donations	Previous blood donors	182 (70.2%)	155 (85.2%)	27 (14.8%)	X^2^(1.259) = 51.261, Df = 1	p-value = <0.0005
	No previous blood donations	77 (29.8%)	32 (41.6%)	45 (58.4%)		

Among participants who are in a relationship, 49.3% (n=102) reported that they are unaware of their partner's blood type. Variables like sex, occupation, and nationality showed no correlation with this knowledge. Age exhibited a borderline significance in relation to knowledge of the partner's blood type [X²(3,207)=7.550, p=0.056]. Those possessing a bachelor's degree or higher displayed notable awareness of their partner's blood type [X²(1,207)=15.096, p<0.0005].

In terms of universal donors and recipients, only 11.6% (n=30) correctly identified O-negative as the universal donor, and 15.5% (n=40) correctly identified AB-positive as the universal recipient. A significant proportion admitted lacking any knowledge of what is a universal donor 40.3% (n=104) or a universal recipient 60.5% (n=156).

Views and practices

**Figure 1:**
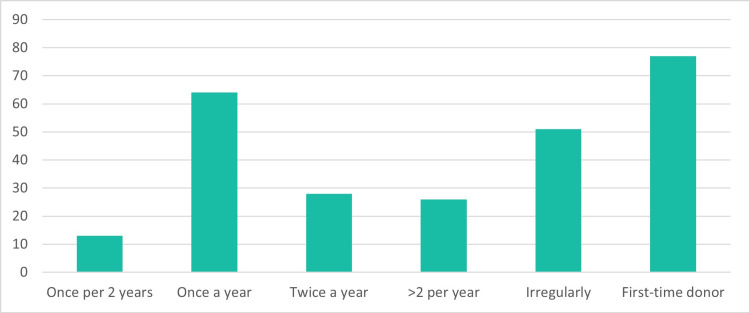
Frequency of blood donation among participants The Y-axis in the figure indicates the number of participants. The total number of studied participants is 259

A majority, 70.3% (n=182) of participants, had a history of at least one blood donation in the past. In Figure 1, the frequency of blood donation among participants is outlined. The majority of participants reported donating blood once a year (24.7%, n=64), while a substantial proportion donated irregularly (19.7%, n=51). First-time donors accounted for 29.7% (n=77) of the participants. Additionally, 10.8% (n=28) donated blood twice a year, and 10.0% (n=26) donated more than twice a year. Those who donated once every two years constituted 5.0% (n=13) of all participants.

**Figure 2:**
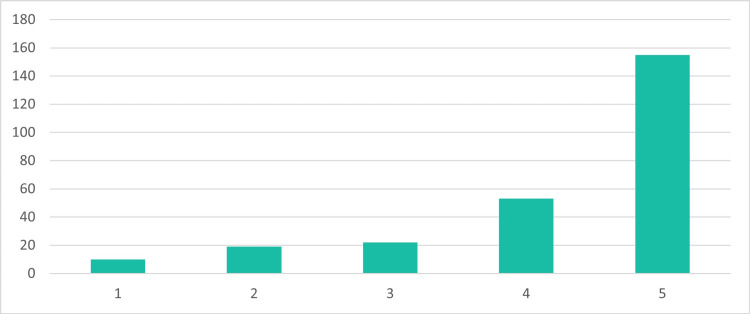
Perceived accessibility of blood donation in the UAE 1: not accessible, 2: slightly accessible, 3: moderately accessible, 4: very accessible, 5: easily accessible The Y-axis in the figure indicates the number of participants. The total number of studied participants is 259

Figure 2 illustrates participants' perceptions of the accessibility of blood donation in the UAE. The majority rated accessibility favorably, with 59.8% (n=155) giving the highest rating (5), while 20.5% (n=53) rated it as a 4. Ratings of 3, 2, and 1 accounted for 8.5% (n=22), 7.3% (n=19), and 3.9% (n=10) of the participants, respectively.

**Figure 3:**
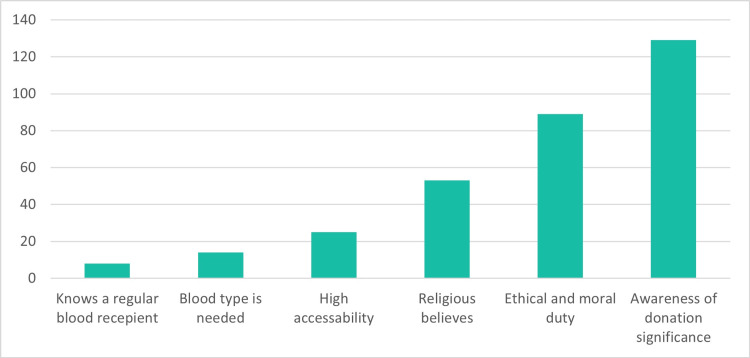
Motives driving blood donation among participants The Y-axis in the figure indicates the number of participants. The total number of studied participants is 259

Motivations for blood donation are depicted in Figure 3. The most prevalent motive was awareness of the significance of blood donation, with 41% (n=129) of participants expressing this altruistic commitment. Ethical and moral duty motivated 28% (n=89) of participants, while 17% (n=53) cited religious beliefs as a driving factor. The need for their blood type (4%, n=14) and knowing a regular blood recipient (3%, n=8) were less frequently reported motives. The study also highlighted that 8% (n=25) of participants were motivated by the high accessibility of blood donation.

Sources of information

The predominant source of participants' knowledge of their blood type was identified as blood typing conducted prior to donation, with 44.7% (n=116) indicating that they became aware of their blood type after donating blood. Other significant sources included former education, 30.9% (n=80), information from family and friends, 28.2% (n=73), social media, 24.7% (n=64), and healthcare providers, 20.5% (n=53). In contrast, books/magazines and TV shows/movies were less frequently consulted, reported by 9.4% (n=24) and 2.5% (n=16) of participants, respectively.

## Discussion

The study revealed diverse patterns in blood donation practices and blood type awareness among participants. The distribution of blood types highlighted a prevalence of O+ and noted the rarity of AB- and O-blood types. Demographic associations revealed that age, sex, marital status, and occupation were not significantly linked to blood type awareness, but nationality, educational level, and history of previous blood donations exhibited a significant association. The frequency of blood donation displayed a balanced distribution between first-time donors and regular contributors, indicating effective donor retention and successful outreach to newcomers. Perceived accessibility was generally high, indicating a positive attitude toward blood donation in the UAE. Motivations varied, with a significant portion driven by awareness of the significance of blood donation and a sense of ethical and moral duty.

Influence of sample demographics on study generalizability

The study strictly adhered to the guidelines adopted by blood banks for donation eligibility, thus only including participants over the age of 18. Notably, the majority of the participants fell within the age bracket of 30s and 40s, which is reflective of the UAE's population composition, which is dominated by labor forces and non-locals [14]. Consequently, males significantly outnumbered females, aligning with the increased male-to-female ratio in the UAE, as most expats are males [14]. This might have been further exacerbated by the increased rates of anemia in females, which is a contraindication for blood donation [18]. In terms of nationalities, the study found biases linked to the location of blood donation campaigns; for instance, a campaign next to the Embassy of Sri Lanka resulted in a disproportionate number of Sri Lankan participants. These demographic factors have implications for both the generalizability of the findings and the development of targeted interventions.

O-positive emerged as the predominant blood type in the study sample, which is consistent with what has been reported by multiple other studies [19,20]. However, that might be partially attributable to the blood bank's targeted outreach to registered donors of this type.

Among locals, higher awareness levels were observed, possibly due to better access to advanced healthcare facilities. Consistent with the literature, educational level correlated positively with blood type knowledge [21]. Age revealed borderline significance in its relation to spousal blood type awareness, potentially ascribable to the lack of experience among younger couples and diminished awareness in older cohorts. Alarmingly, there was a lack of awareness concerning the risks of transfusion mismatches and the role of universal donors, indicating a pressing need for educational interventions.

Institutional support for blood donation in the UAE

The UAE has consistently displayed an encouraging and positive attitude when enticing citizens to donate blood. This is highlighted by our study, as most participants considered the accessibility of blood donation in the UAE to be high. Health authorities spanning all emirates provide the option to register as a blood donor through phone applications and websites. Blood donation can be facilitated not just in designated centers but also through mobile blood banks that visit high-traffic public spaces and university campuses, thereby minimizing the barriers that may prevent potential donors from turning their intention into action.

Emirates Health Services, the federal healthcare system in the UAE, provides eight blood donor sites spanning all of the northern Emirates. The Department of Health in Abu Dhabi has partnered with the UAE Volunteer Platform to broaden the donor base, specifically targeting young adults [22]. Donors receive accredited volunteer hours and certificates as incentives, a strategy that has seen success in increasing donor numbers [23]. Data from Abu Dhabi Blood Bank Services confirms an exponential and steady rise in annual donors, attesting to the effectiveness of these initiatives [23]. In the year 2012, Dubai Health founded an annual campaign under the name “My Blood, for My Country." Every year, the campaign is launched by Dubai Blood Donation Center (DBDC), visiting a multitude of sites around the emirate. By 2020, DBDC accounted for approximately 50% of total blood donated across the country [24].

Public health context: UAE's higher demand for blood donations

In the UAE, it is reasonable to infer that the need for blood transfusions is uniquely high, owing to a high prevalence of thalassemia cases and road traffic accident-related trauma injuries. Although premarital screening for hemoglobinopathies is controlling the number of thalassemia cases in the UAE [18], the prevalence of the disease among UAE nationals remains one of the highest in the world [25,26]. With transfusion therapy being one of the mainstay treatments for thalassemia, that poses a high demand for blood transfusion units [27]. Additionally, the incidence of road traffic accidents contributes substantially to trauma-related injuries, further escalating the need for readily available blood products [28]. These unique epidemiological and public health challenges position the UAE as a critical landscape for innovative blood donation strategies and healthcare interventions.

Proposed strategies for enhancing blood donation rates

The elevated rate of previous donors (70.3%) within our study population suggests the effectiveness of current blood donation optimization strategies in the UAE. Initiatives such as mobile applications for blood donation, portable donation centers, annual campaigns, and donor incentives might have positively impacted the blood supply. However, this study aims to propose additional measures to attract new donors and foster sustained relationships with existing donors, particularly those with less common or critical blood types like O-negative.

Firstly, incorporating blood type information on personal identification documents, such as the Emirates ID, could incentivize blood donation. As supported by this study and existing literature, awareness of one's blood type is correlated with an increased likelihood of donation. To complement this, we propose an educational campaign focused on blood type compatibility and the risks associated with transfusion mismatches. Tailored invitations for blood donation could be integrated into the Emirates ID renewal process, utilizing culturally relevant multimedia to engage potential donors. According to our data, a considerable majority (95.7%) expressed interest in having blood types documented on official identification.

Secondly, a notification system could be implemented to alert registered donors of impending blood shortages and to schedule donation appointments. This system could also notify donors when their donation eligibility period has lapsed, prompting them to donate again. A subscription service could be integrated for those willing to donate blood regularly, where donors can opt for reminders at a frequency of their choosing.

These proposed interventions serve dual purposes: not only do they aim to augment the donor pool, but they also strive to enhance the quality of patient care. Existing literature indicates that, while universal donor blood types may be used in critical and urgent circumstances, it is preferable to match patients with their specific blood types to mitigate the risk of complications [29]. Consequently, these strategies aim to both alleviate shortages during crisis periods and improve patient outcomes by enabling more precise blood type matching for transfusions.

Limitations

This study bears several limitations that warrant consideration. As a cross-sectional study, it captures data at a single point in time and thus cannot establish causality. Furthermore, the sample is derived from a blood bank that excluded certain individuals based on blood type abundance or ineligibility criteria, such as low hemoglobin levels. This selection bias led to unequal gender representation, particularly underrepresenting females due to a higher prevalence of anemia in this demographic. Future research should consider alternative settings outside of blood banks to better represent the diverse UAE population.

Recommendations

In light of the findings, we recommend two key interventions to enhance blood donation rates in the UAE. First, blood type information should be incorporated into personal identification documents, such as the Emirates ID. This measure, supplemented by an educational campaign on blood type compatibility, aims to incentivize donation by raising awareness and facilitating tailored donor invitations during the ID renewal process. Second, an automated notification system is proposed to alert donors of impending blood shortages and their donation eligibility, with an optional subscription feature for regular reminders. These recommendations serve to expand the donor pool and foster sustained relationships with donors, thereby not only mitigating blood shortages but also improving patient outcomes through more precise blood type matching.

## Conclusions

Most participants were able to correctly identify their blood type; awareness was associated with Emirati nationality, higher educational achievement, and a history of previous blood donation. The high perceived accessibility of blood donation, along with the observed prevalence of previous blood donations among participants, offers valuable feedback on the effectiveness of ongoing initiatives in the UAE for blood donation. Our study identifies target demographics and motivational triggers, offering valuable insights for optimizing blood donation initiatives in the UAE. The scarcity of O-blood type among participants, coupled with its critical role in emergency situations, further highlights the need for initiatives targeting increased awareness and donation of this blood type. The study suggests practical measures, like incorporating blood type information on Emirates IDs and employing notification systems to encourage donation, especially during shortages. Such strategies could effectively enhance blood donation rates and improve the safety and efficacy of blood transfusions in the UAE, aligning with the region's unique public health needs and demographic profile.

## Appendices

The following questionnaire was developed and employed for data collection in this study. It consists of 21 items organized into five sections, covering demographics, knowledge and awareness, practices and views, sources of information, and the participant's blood type as reported by the blood bank.

Identifiers

1- Participant's ID:

2- Blood group as reported by the blood bank: (A +, A -, B +, B -, AB +, AB -, O +, O -).

Demographics

3- Age: (in years).

4- Sex: (male, female).

5- Nationality: (local, non-local).

- If "non-local", specify:

6- Social status: (in a relationship, single).

7- Education achieved: (elementary school, middle school, high school, technical school, college or diploma degree, bachelor's degree, postgraduate degree).

8- Occupation: (medical field, nonmedical field, unemployed).

Knowledge and awareness

9- Are you aware of your own blood group? (yes, no/unsure).

- If "no/unsure", go to question 11.

10- What is your blood group? (A +, A -, B +, B -, AB +, AB -, O +, O -).

11- *If social status is “single,” go to question 13*. Are you aware of your partner’s blood group? (yes, no/unsure).

- If "no/unsure", go to question 13.

12- What is your partner’s blood group? (A +, A -, B +, B -, AB +, AB -, O +, O -).

13- Are you aware of the concept of blood compatibility? (yes, no).

- Explanation: If someone is sick and needs a blood transfusion, the blood type given to them should be compatible with their own blood type.

14- Do you know which blood type is considered as the “universal blood donor”? (A +, A -, B +, B -, AB +, AB -, O +, O -, I do not know).

- Explanation: Which blood type can be given to people of any blood group without causing adverse events related to compatibility?

15- Do you know which blood type is considered as the “universal recipient”? (A +, A -, B +, B -, AB +, AB -, O +, O -, I do not know).

- Explanation: People with which blood type can receive blood of any blood group without experiencing adverse events related to compatibility.

Practices and views

16- Is this your first time donating blood? (yes, no), If "yes," go to question 18.

17- How frequently do you donate blood? (irregularly, once every other year, once a year, twice a year, more than twice a year).

18- On a scale from 1 to 5, how accessible do you find blood donation in the UAE (1 is not accessible and 5 is easily accessible)? (1, 2, 3, 4, 5).

19- What are the motives driving you to donate blood? (check all that applies): (blood donation center is accessible, Knows someone who requires regular transfusions, aware of the significance of blood donation, their blood type is needed, ethical and moral duty, religious beliefs, other).

- If "other," specify:

20- What is your opinion on reporting your blood type on your official documents (e.g. Emirates ID): (yes, no).

Sources of information

21- From which sources have you obtained information regarding blood types and blood donation? (check all that applies): (my healthcare provider, school/university, family/friends, books/magazines, social media, television shows/movies, other).

- If "other," specify:
